# Blue light directly modulates the quorum network in the human pathogen *Acinetobacter baumannii*

**DOI:** 10.1038/s41598-021-92845-1

**Published:** 2021-06-28

**Authors:** Marisel Romina Tuttobene, Gabriela Leticia Müller, Lucía Blasco, Natalia Arana, Mónica Hourcade, Lautaro Diacovich, Pamela Cribb, María Tomás, Carlos Gabriel Nieto-Peñalver, María Alejandra Mussi

**Affiliations:** 1 Centro de Estudios Fotosintéticos y Bioquímicos (CEFOBI-CONICET), 2000 Rosario, Argentina; 2 Microbiology Department-Biomedical Research Institute A Coruña (INIBIC), Hospital A Coruña (CHUAC), University of A Coruña (UDC), A Coruña, Spain; 3 Gas Chromatography/Mass Spectrometry Laboratory, Universidad Nacional de Rosario, Rosario, Argentina; 4 Instituto de Biología Celular y Molecular de Rosario (IBR-CONICET), Ocampo y Esmeralda, 2000 Rosario, Argentina; 5 PROIMI, CONICET (Planta Piloto de Procesos Industriales Microbiológicos), Av. Belgrano y Pje. Caseros, Tucumán, Argentina; 6 Instituto de Microbiología, Facultad de Bioquímica, Química y Farmacia, Universidad Nacional de Tucumán, Tucumán, Argentina

**Keywords:** Microbiology, Bacteria, Microbial communities

## Abstract

Quorum sensing modulates bacterial collective behaviors including biofilm formation, motility and virulence in the important human pathogen *Acinetobacter baumannii*. Disruption of quorum sensing has emerged as a promising strategy with important therapeutic potential. In this work, we show that light modulates the production of acyl-homoserine lactones (AHLs), which were produced in higher levels in the dark than under blue light at environmental temperatures, a response that depends on the AHL synthase, AbaI, and on the photoreceptor BlsA. BlsA interacts with the transcriptional regulator AbaR in the dark at environmental temperatures, inducing *abaI* expression. Under blue light, BlsA does not interact with AbaR, but induces expression of the lactonase *aidA* and quorum quenching*,* consistently with lack of motility at this condition. At temperatures found in warm-blooded hosts, the production of AHLs, quorum quenching as well as *abaI* and *aidA* expression were also modulated by light, though in this case higher levels of AHLs were detected under blue light than in the dark, in a BlsA-independent manner. Finally, AbaI reduces *A. baumannii*'s ability to kill *C. albicans* only in the dark both at environmental as well as at temperatures found in warm-blooded hosts. The overall data indicate that light directly modulates quorum network in *A. baumannii*.

## Introduction

*Acinetobacter baumannii *has been recognized by the World Health Organization (WHO) as one of the most threatening bacterial pathogens deserving urgent action^1^. The global menace from this pathogen arises from its high ability to develop antibiotic resistance as well as its outstanding ability to persist in the environment, which lead to the rapid emergence and spread of multidrug-resistant clinical isolates^2,3^. This makes the need for understanding the mechanisms of resistance and virulence critical.

Quorum sensing (QS) has been shown to modulate bacterial collective behaviors such as biofilm formation, motility, virulence, and even drug resistance mechanisms in a number of bacteria including *A. baumannii*^4–7^. Quorum sensing is a cell–cell communication system employed by bacteria to coordinate the expression of specific genes as a function of population density. In most Gram-negative bacteria, QS is mediated via the synthesis, release and detection of diffusible signaling molecules such as the *N*-acyl-homoserine lactones (AHLs)^8^. *A. baumannii* and related pathogenic *Acinetobacter* spp. possess a canonical Gram-negative LuxR/LuxI QS system, consisting of an AHL synthase (AbaI) and a transcriptional regulator (AbaR) that is activated on binding an AHL, leading ultimately to diverse cellular responses^9^. AbaR bound to AHL triggers the production of more AHLs in a positive feedback loop manner. The complete genome sequencing of *A. baummannii* ATCC 17978 revealed that AbaI may be the sole participant for the production of AHLs with varying chemical structures^5^. Recently, the product of the *abaM* gene, which is located between the *abaR* and *abaI* genes in the *A. baumannii* chromosome, has been shown to play a central role in QS negative regulation^10^. Lately, disruption of QS has emerged as an anti-virulence strategy with important therapeutic potential^11^. Quorum quenching (QQ) refers to all processes involved in the disturbance of QS^12^. QQ molecular actors are diverse in nature (enzymes, chemical compounds), mode of action (QS-signal cleavage, competitive inhibition, etc.) and targets, as all main steps of the QS pathway that are synthesis, diffusion, accumulation and perception of the QS signals may be affected^13^.

Some years ago, we have recognized a new aspect of *A. baumannii*'s physiology: its ability to perceive light and respond to this stimuli modulating different traits related to persistence at environmental temperatures^14^. In fact, light modulates biofilm formation, motility, killing of competitors, phenylacetic acid, trehalose and acetoin metabolisms, iron uptake, antioxidant enzymes production, antibiotic susceptibility and tolerance to antibiotics, at environmental temperatures^14–21^. Regulation by light of many of these traits depends on the photoreceptor BlsA, which is operative in the 18–24 °C low-moderate temperature range^14–16^. In particular, *blsA* transcript levels were significantly reduced at temperatures higher than 25 °C, in agreement with undetectable BlsA protein levels in the cell at 26 °C and higher temperatures. Also, quantum yield of photo-activation of BlsA (lBlsA) between 14 and 37 °C, showed that BlsA photoactivity is greatly compromised at 25 °C and absent above 28 °C^16^. We have shown that BlsA interacts with and antagonizes the action of Fur or AcoN transcriptional repressors, allowing expression of their regulated genes only in the dark or in the presence of blue light, respectively^18,20,21^. More recently, we have provided evidence indicating that ESKAPE pathogens such as *Staphylococcus aureus*, *Pseudomonas aeruginosa*, and *Acinetobacter baumannii*, also perceive and respond to light at temperatures found in warm-blooded hosts, regulating virulence in an epithelial infection model as well as important pathogenicity determinants, which could have implications in human infections^22^.

In Tuttobene et al*.*^21^, we observed that both quorum and light integrate signals into acetoin catabolism through the AcoN transcriptional regulator in *A. baumannii*. This connection prompted us to systematically explore the possible relation between light and quorum in *A. baumannii*. We decided to focus on two community behaviors such as motility and biofilm formation shown to depend on quorum sensing, and which are also modulated by light^14,16^. Our results show that there is a fine tuning of quorum sensing vs. quenching activities dictated by modulation of the expression of AHL synthases and lactonases by light, integrating also a temperature signal, which results in the differential production of AHLs in response to illumination and temperature. Overall, in this work we provide evidence indicating that light directly modulates the quorum network in *A. baumannii*.

## Results

### *AbaI* is a direct or indirect component of the light signaling cascade in *A. baumannii* at environmental temperatures

**Figure 1:**
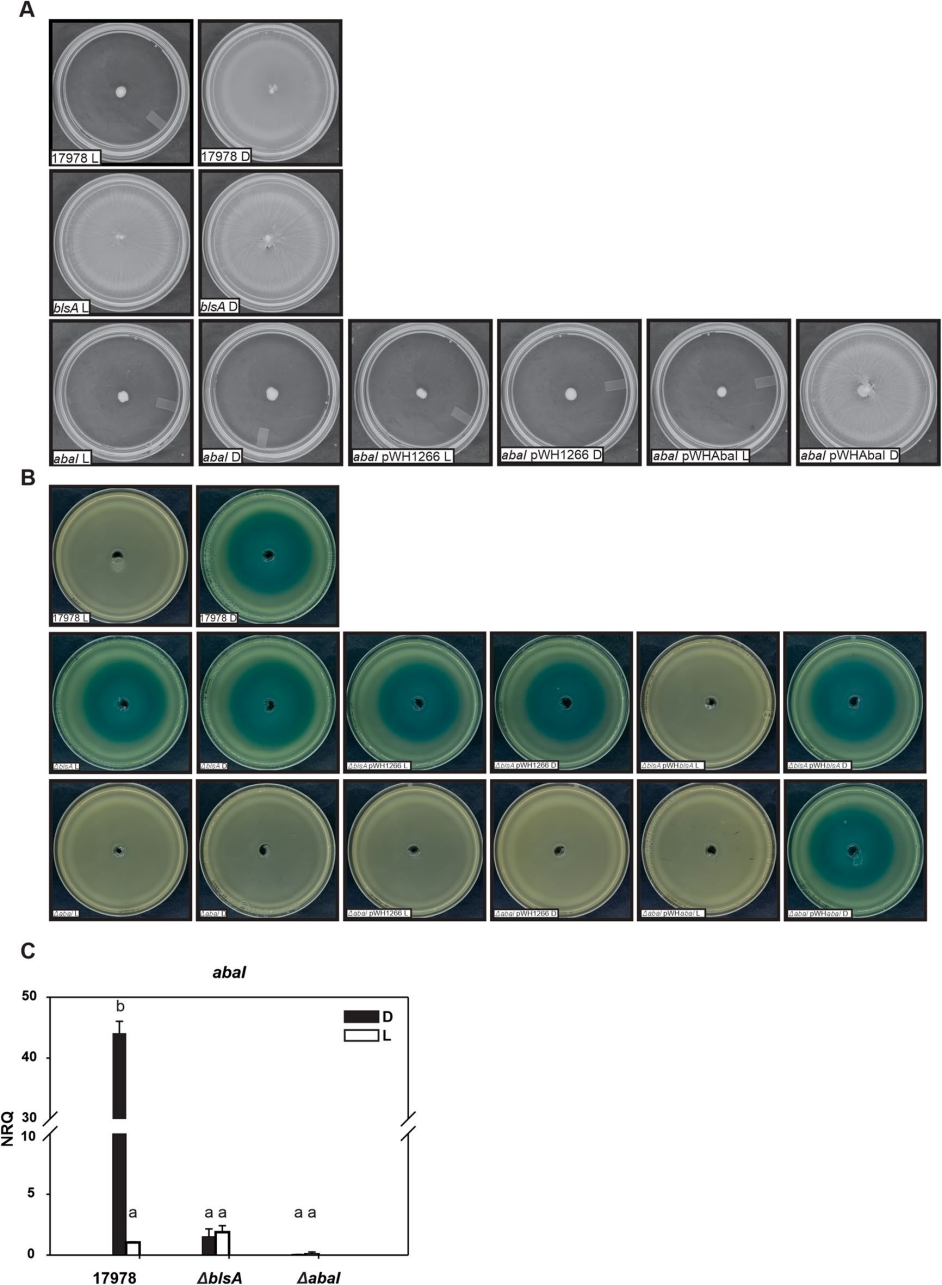
Light modulates AHLs production through BlsA and AbaI in cells recovered from motility plates of *A. baumannii* at environmental temperatures. (**A**) Cells of the parental strain ATCC 17978, the isogenic *ΔblsA* and *ΔabaI* mutants, as well as this mutant harboring the empty pWH1266 plasmid or the *abaI*-complementing plasmid pWHAbaI were inoculated on the surface of motility plates. Plates were inspected and photographed after incubation in darkness (D) or in the presence of blue light (L) at 23 °C. Representative results of three independent experiments are shown. (**B**) Supernatants recovered from motility plates of the indicated strains incubated under blue light or in the dark at 23 °C were filtered sterilized, and amounts normalized to a OD_660_ = 1.5 were then loaded in a central well of biosensor-inoculated LB plates. The *A. tumefaciens* NT1 (pZLR4) biosensor produces 5,5′-dibromo-4,4′-dichloro-indigo as a result of the presence of C_6_–C_12_ AHLs in the supernatants. Plates were inspected and photographed after incubation in darkness (D) at 30 °C for 24 h. Representative results of three independent experiments are shown. (**C**) Estimation by qRT-PCR of the expression levels of the gene coding for an acyl homoserine lactone synthase, *abaI* in cells recovered from motility plates inoculated with ATCC 17978 wild-type, *∆blsA* and *∆abaI* incubated at 23 °C under blue light (L) or in the dark (D). Shown are the mean and standard deviation of normalized relative quantities (NRQ). Significant differences determined by ANOVA followed by Tukey’s multiple comparison test (*p* < 0.05) are indicated by different letters. Shown are representative results of three independent experiments.

*A. baumannii* ATCC 17978 moved covering the whole plate in the dark, while its motility was inhibited in the presence of light at 23 °C (Fig. 1A)^14^. The mutant in the only traditional photoreceptor encoded in the *A. baumannii* genome, *blsA,* lost photoregulation at this temperature, as motility covered the whole plates under both illumination conditions (Fig. 1A)^14^. Several studies have shown that *A. baumannii abaI* mutants are deficient in motility and biofilm formation^5,9,23,24^. Figure 1A shows that in the *ΔabaI* mutant motility was abolished both under blue light and in the dark at 23 °C, as also was motility in the *ΔabaI* mutant harboring the pWH1266 plasmid. In contrast, the *ΔabaI* mutant containing plasmid pWHAbaI, which expresses a wild type copy of *abaI* directed by its own promoter, rescued the wild type phenotype resulting in motility only in the dark but not under blue light (Fig. 1A), integrating thus a light signal.

The results presented here show that AbaI is required for motility at environmental temperatures, and that it is a direct or indirect component of the light signaling cascade.

### Light modulates the production of AHLs in motility and biofilms through BlsA and AbaI in *A. baumannii* at moderate temperatures

To study if quorum sensing is modulated by light in *A. baumannii*, we first evaluated the effect of light in AHLs production on quorum-dependent processes by using different bacterial biosensors. AHL biosensors are bacterial strains in which the quorum cascade is activated upon complementation with exogenous AHLs, leading to a clearly evident phenotype such as pigment production.

For this purpose, filtered sterilized supernatants were generated from cultures recovered from motility plates incubated under blue light or in the dark at 23 °C*.* The amounts of supernatants were normalized to bacterial optical density OD_660_ = 1.5 (see “Methods” for details), and then located in the center wells of biosensor-inoculated LB plates.

The *Agrobacterium tumefaciens* NT1 (pZLR4) AHL biosensor^25,26^, responds to AHLs of chain lengths ranging from C_6_ to C_12_^27^. The supernatant recovered from ATCC 17978 motility plates incubated in the dark stimulated expression of the β-galactosidase gene from the quorum-directed promoter in *A. tumefaciens*, indicating the presence of AHLs in this condition (Table 1; Fig. 1B). In contrast, the absence of β-galactosidase activity in samples from motility plates of the wild type strain under light conditions, reflected the absence of detectable amounts of AHLs in this condition (Table 1; Fig. 1B). The *ΔblsA* mutant as well as the *ΔblsA* mutant harboring the empty pWH1266 plasmid produced AHLs when incubated both under blue light or in the dark, as similar haloes were produced in both conditions (Table 1; Fig. 1B). On its side, the *ΔblsA* mutant harboring plasmid pWHBlsA, which expresses a wild type copy of the *blsA* gene directed by its own promoter, recovered the wild type phenotype stimulating β-galactosidase expression only when supernatants from motility plates incubated in the dark but not under blue light were added to *A. tumefaciens* plates (Table 1; Fig. 1B). The supernatants obtained from the *ΔabaI* mutant as well as from the *ΔabaI* mutant harboring pWH1266 motility plates under blue light and in the dark both failed to induce β-galactosidase expression (Table 1; Fig. 1B). This indicates absence of detectable amounts of AHLs in the *ΔabaI* mutant, which is the expected results since the AHL synthase is absent. The *ΔabaI* mutant harboring pWHAbaI, in contrast, restored the behavior observed for the wild type strain, *i.e.*, only the supernatant recovered from *ΔabaI* pWHAbaI motility plates in the dark stimulated β-galactosidase expression in *A. tumefaciens*, but not that recovered from plates incubated under blue light (Table 1; Fig. 1B).

As controls, bioassays performed with *A. tumefaciens* NT1 (pZLR4) in the presence of different standards (C8-, C10-, and C12-HSL as well as 3OHC8-, 3OHC10- and 3OHC12-HSL) show that all of them, including the hydroxylated and non-hydroxylated forms, could be detected under our experimental conditions, though to different extents (Supplementary Figure S3)^28^.

Similar experiments performed using the *Chromobacterium violaceum* VIR07 or the *Chromobacterium subtsugae* CV026 biosensors, which synthesize violacein in the presence of long-chain or short-chain AHLs^28,29^, respectively, produced similar patterns as that observed for *A. tumefaciens* NT1 (pZLR4) (Table 1; Supplementary Figure S1). As controls, bioassays performed with these biosensors in the presence of different standards (C8-, C10-, and C12-HSL as well as 3OHC8-, 3OHC10- and 3OHC12-HSL) show that all of them, including the hydroxylated and non-hydroxylated forms, could be detected by *C. violaceum* VIR07 under our experimental conditions, though to different extents (Supplementary Figure S3). On the contrary, *C. subtsugae* CV026 only detected C8-AHL (Supplementary Figure S3).

The overall results thus indicate that the production of AHLs is regulated by light in motility in *A. baumannii* at moderate temperatures*,* in a BlsA and AbaI-dependent manner (Table 1; Fig. 1B).

We also assayed the effect of light on AHL production in another quorum-dependent phenotype, biofilm formation. For this purpose, filtered sterilized supernatants were generated from cultures recovered from biofilm tubes incubated under blue light or in the dark at 23 °C*.* The amounts of supernatants were normalized to bacterial optical density OD_660_ = 0.5 (see “Methods” for details), and then located on wells in the center of biosensor-inoculated LB plates.

As previously described and as shown in Supplementary Figure S2A, biofilm formation is modulated by light in *A. baumannii* at environmental temperatures, in a BlsA-dependent manner^14^. In particular, biofilms are significantly produced in the dark while inhibited in the presence of light in ATCC 17978. The photorregulation is lost in the *blsA* mutant, and the wild type phenotype is rescued in the *blsA* complementing strain (Supplementary Figure S2A). Biofilms are not significantly produced in the *abaI* mutant, while the *abaI* complementing strain rescued the wild type phenotype indicating that photoregulation also depends on AbaI (Supplementary Figure S2A).

Supplementary Figure S2B shows that supernatants recovered from ATCC 17978 biofilms incubated in the dark significantly stimulated the production of violacein in *C. subtsugae* CV026, indicating the presence of AHLs (Table 1; Supplementary Figure S2B). In contrast, the absence of pigment in the case of supernatants recovered from ATCC 17978 biofilms incubated under blue light, reflected the absence of detectable amounts of AHLs (Table 1; Supplementary Figure S2B). The *ΔblsA* mutant as well as the *ΔblsA* mutant harboring pWH1266 produced similar levels of AHLs both when biofilm tubes were incubated under blue light or in the dark, as similar haloes of violacein were produced at both conditions (Table 1; Supplementary Figure S2B). On the contrary, the *ΔblsA* mutant harboring pWHBlsA rescued the wild type phenotype. In fact, the production of violacein was significantly stimulated only when supernatants recovered from biofilms in the dark but not under blue light were added to *C. subtsugae* CV026 plates (Table 1; Supplementary Figure S2B). It is noteworthy that incubation of *C. subtsugae* CV026 with the supernatant recovered from *ΔblsA* pWHBlsA biofilms in the dark, resulted in higher violacein haloes than when supernatants recovered from the wild type strain were incubated in the dark. This effect can be explained considering the extra *blsA* gene doses in the former, as has already been observed before^14^. The supernatants obtained from *ΔabaI* or *ΔabaI* mutant harboring pWH1266 biofilms incubated under blue light or in the dark at 23 °C failed to induce violacein production (Table 1; Supplementary Figure S2B). This indicates absence of detectable amounts of AHLs production in the *ΔabaI* mutant, as expected. The *ΔabaI* mutant harboring pWH*abaI*, in contrast, rescued the wild type phenotype (Table 1; Supplementary Figure S2B), i.e., only supernatant recovered from *ΔabaI* pWHAbaI biofilms incubated in the dark significantly stimulated the production of violacein in *C. subtsugae* CV026, but not that recovered from plates incubated under blue light. Again, the supernatant recovered from *ΔabaI* pWHAbaI motility plates incubated in the dark, resulted in higher violacein haloes than when supernatants recovered from the wild type strain were incubated in the dark. Overall, these results thus show that the production of short chain AHLs is modulated by light in biofilms at moderate temperatures in *A. baumannii* ATCC 17978, in a BlsA and AbaI-dependent manner.

Similar experiments performed using the long-chain AHLs biosensor *C. violaceum* VIR07, produced similar pattern as that observed for C. *subtsugae* CV026 (Table 1; Supplementary Figure S2C), indicating that the production of long chain AHLs is regulated by light in biofilms in *A. baumannii,* in a BlsA and AbaI-dependent manner at moderate temperatures (Table 1; Supplementary Figure S2C).

Finally, AHL production (Figs. 1; Supplementary Figures S1, S2) follows the motility as well as the biofilm formation pattern (Fig. 1A; Supplementary Figure S2A).

### Light modulates ***abaI*** expression at 23 °C through the BlsA photoreceptor

To get insights into the molecular mechanism of light modulation of quorum sensing, we studied the expression of *abaI* in cells recovered from motility plates incubated under blue light or in the dark at 23 °C. Figure 1C shows that expression of *abaI* is approximately 38 fold higher in the dark than in the presence of light, indicating that it is modulated by light in ATCC 17978. In the *Δbls*A mutant, *abaI* expression levels were similar between light and dark conditions, and comparable to wild type levels in the presence of light (Fig. 1C), showing loss of photoregulation. As expected, expression of *abaI* in the *ΔabaI* mutant was null or negligible (Fig. 1C). The overall results show that the expression of *abaI* is modulated by light, being induced in the dark at 23 °C through the BlsA photoreceptor. This result is consistent with the higher production of AHLs in the dark in ATCC 17978.

### Light stimulates quorum quenching activity at moderate temperatures in *A. baumannii*

As another approach to study the effect of light on the production of quorum quenching molecules at moderate temperatures, we evaluated whether the supernatants of sonicated cells recovered from motility plates incubated under blue light or in the dark presented quorum quenching activity. For this purpose, these supernatants were incubated in the presence of 2 µg of C8-AHL for 6 h, and then the mixture was loaded on a central well generated in a *C. subtsugae* CV026*-*inoculated LB plate. The extent of inhibition of violacein production compared to the control provides an indication of quorum quenching activity.

**Figure 2:**
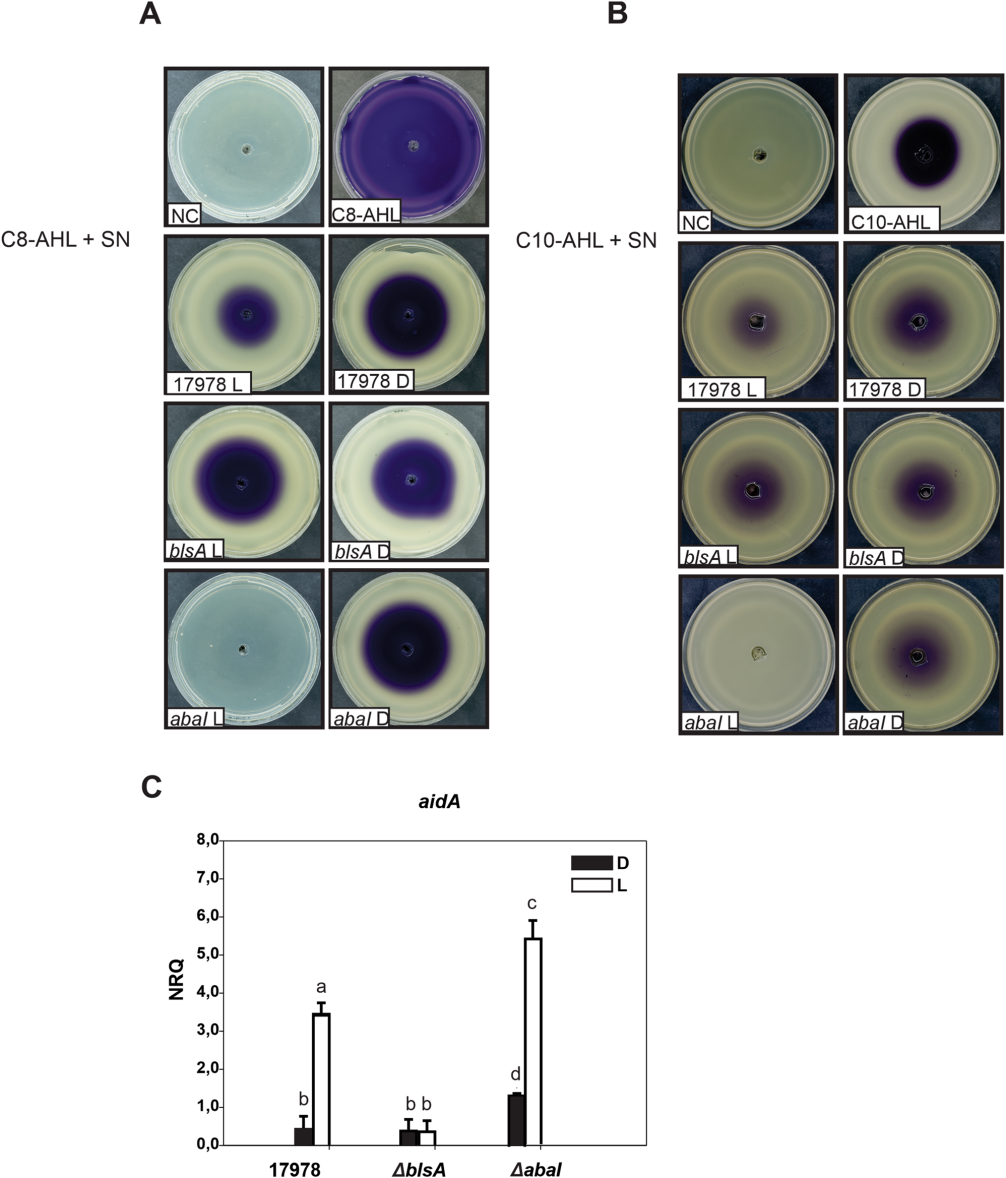
Light modulates quorum quenching activity and *aidA* expression at environmental temperatures in *A. baumannii*. (**A**,**B**) Quorum quenching assays were performed by incubating the post-sonicated supernatants recovered from motility plates of the indicated strains incubated under blue light or in the dark at 23 °C and normalized to OD_660_ = 1.5, with 2 µg of commercial standard for 6 h at 37 °C. After incubation, the mixture was then loaded in a central well of biosensor-inoculated LB plates. The biosensors used are the short-chain AHL biosensor *C. subtsugae* CV026 (**A)** as well as the long-chain AHL biosensor *C. violaceum* VIR07 (**B**). Negative control (NC) was performed by inoculating PBS 1X in the central well of the plates. The positive controls (standards labeled C8-AHL or C10-AHL) were performed by adding 2 µg of each commercial standard. The extent of inhibition of violacein production compared to the control provides an indication of lactonase activity. Plates were inspected and photographed after incubation in darkness (D) at 30 °C for 24 h. Shown are representative results from three independent experiments. (**C**) Estimation by qRT-PCR of the expression levels of the gene coding for a lactonase, *aidA,* in cells recovered from motility plates inoculated with ATCC 17978 wild-type, *∆blsA* and *∆abaI* incubated at 23 °C under blue light (L) or in the dark (D). Shown are the mean and standard deviation of normalized relative quantities (NRQ). Significant differences determined by ANOVA followed by Tukey’s multiple comparison test (*p* < 0.05) are indicated by different letters. Representative results of three independent experiments are shown.

As shown before, the addition of 2 µg of commercial C8-AHL to the central well of *C. subtsugae* CV026-inoculated LB plates stimulated the production of violacein in the whole plate (Fig. 2A). Interestingly, when this fixed amount of standard was mixed with the supernatant of the sonicated wild type cells from motility plates under blue light, violacein production was less produced than when they were incubated in the dark (Fig. 2A). In contrast, when the standard was incubated with supernatants from sonicated *ΔblsA* mutant cells incubated either under blue light or in the dark, similar capability to inhibit violacein production was observed, which was similar to that of the wild type incubated in the dark, showing thus loss of photoregulation of quorum quenching activity (Fig. 2A). Finally, when supernatants from sonicated *ΔabaI* cells recovered from motility plates under blue light at 23 °C were used, no violacein production was observed indicating that the standard was completely degraded (Fig. 2A). On the contrary, when the standard was incubated with supernatants of sonicated *ΔabaI* pWH*abaI* cells from motility plates incubated in the dark at 23 °, *C. subtsugae* CV026 produced an important violacein halo comparable to that of the wild type in the dark (Fig. 2A). These results thus indicate much higher quorum quenching activity under blue light than in the dark in this strain, much higher even than in the wild type strain (Fig. 2A).

Similar experiments performed using the *C. violaceum* VIR07 biosensor and C10-AHL as standard, produced similar patters as *C. subtsugae* CV026 (Fig. 2B), indicating that quorum quenching activity affects long-chain AHLs as well. Overall, we show here that the production of AHL-inactivating activity is modulated by light, in a BlsA-dependent manner. The fact that quorum quenching activity was observed in sonicated supernatants strongly suggest the presence of non-secreted lactonase/s.

### AidA expression is modulated by light at 23 °C through the BlsA photoreceptor

Given that our data provide evidence indicating modulation by light of quorum quenching activity, we next decided to analyze by qRT-PCR whether expression of the gene coding for a recently described lactonase, *aidA*, is responsive to light^30^. AidA has been proposed to degrade 3-oxo-C12-HSL; however, its activity has not been characterized against other AHL. It is possible though that as a lactonase it is able to degrade different types of AHLs^30^.

Our results show that *aidA* expression is approximately 7 folds higher when ATCC 17978 cells were recovered from motility plates incubated under blue light compared to darkness (Fig. 2C). The *ΔabaI* mutant behaved similarly as the wild type strain, showing differential expression of *aidA* in response to light, despite expression levels were much higher both under blue light and in the dark (Fig. 2C). The observed photoregulation depends on BlsA, since the *ΔblsA* mutant was basal and similar under blue light and in the dark (Fig. 2C).

The results obtained here show that expression of *aidA* gene in ATCC 17978 and in the *ΔabaI* mutant is modulated by light through the BlsA photoreceptor at environmental temperatures. This result is consistent with the modulation by light of quorum quenching activity shown using biosensors (Fig. 2A,B).

### Detection of Acyl-HSLs by TLC and GC-mass spectrometry at 23 °C

**Figure 3:**
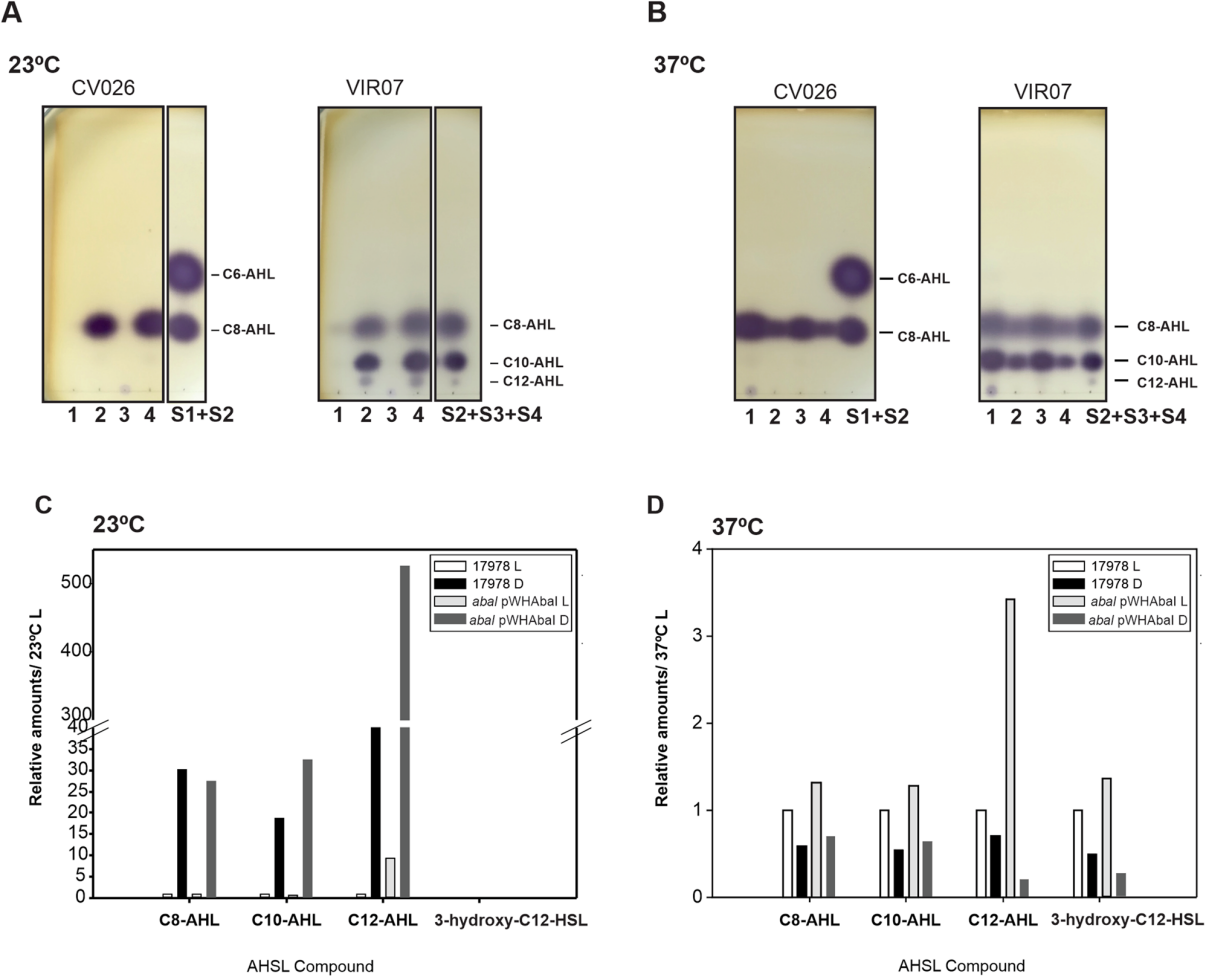
TLC and GC-mass spectrometry of acyl-HSLs extracted from *A. baumannii* biofilms. (**A**,**B**) AHLs extracts were chromatographed on C_18_ reversed-phase TLC, developed with 60% methanol, and the spots were visualized with the *C. subtsugae* CV026 or *C. violaceum* VIR07 reporter strains. Samples correspond to AHLs extracts of *A. baumannii* ATCC 17978 biofilm cultures incubated at 23 °C (**A**) or 37 °C (**B**) under blue light (lane 1) and in the dark (lane 2); or the *abaI* mutant harboring pWHabaI under blue light (lane 3) and in the dark (lane 4). S1: C6-AHL, S2: C8-AHL, S3: C10-AHL and S4: C12-HSL. S2 + S3 + S4: a mixture of the S3, S4 and S5 standards. Results shown are representative of 2 independent experiments. (**C**,**D**) AHL extracts were dissolved in 100 µl HPLC-grade acetonitrile and silylated in a nitrogen atmosphere (1 h at 80 °C). One μl of derivatized sample was injected (Split 1:10) into the GC-mass spectrometer. For each condition (23 or 37 °C), quantitation ion's areas of each AHL were relativized to the one obtained in the 17978 sample incubated under blue light condition. Results shown are representative of 2 independent experiments.

We next extracted the AHLs fraction from biofilms supernatants of the wild type *A. baumannii* ATCC 17978 strain, and its isogenic mutant *abaI* harboring the pWHAbaI plasmid grown under blue light and in the dark at 23 °C, and separated them by TLC (Fig. 3A). At 23 °C, the presence of AHLs was observed only in the dark but not under blue light, when TLCs were developed using *C. subtsugae* CV026 for short-chain AHLs, for both *A. baumannii* strains (Fig. 3A). According to the standards, the spots observed were compatible with C8-HSL (Fig. 3A; Supplementary Figure S3). When equivalent TLCs were developed with *C. violaceum* VIR07 for long-chain AHLs, three different spots were observed in higher levels in the dark respect to blue light, for both *A. baumannii* strains (Fig. 3A). In this case, spots were compatible with C8-AHL, C10-AHL and C12-AHL standards. Figure 3C shows GC–MS results, in which the quantitation ion’s areas of each AHL were relativized to the one obtained in 17978 at 23 °C under blue light. As observed in Fig. 3C, no hydroxylated AHLs were detected at 23 °C, despite C8-C10 and C12-AHLs were detected. Interestingly, and consistently with the biosensor assays as well as with TLCs, the presence of C8, C10 and C12-AHLs was detected in higher amounts in the dark respect to blue light conditions both for the wild type as well as for the *abaI* mutant harboring pWHAbaI (Fig. 3C).

### AbaI is involved in modulation by light of *A. baumannii* ATCC 17978 ability to kill *C. albicans tup1* at environmental temperatures

To study the role of quorum sensing and light in the ability of *A. baumannii* to kill *C. albicans* at moderate temperatures, ATCC 17978 or its isogenic *ΔabaI* mutant and derived strains were co-incubated with *C. albicans tup1* at 23 °C, and the survival of *C. albicans* was recorded as described in^14^. For killing assays, *C. albicans tup1* mutants were used, as it was previously shown that *A. baumannii* is able to attach to and kill *tup1* filaments but not the parental SC5314 yeast cells^31^.

**Figure 4:**
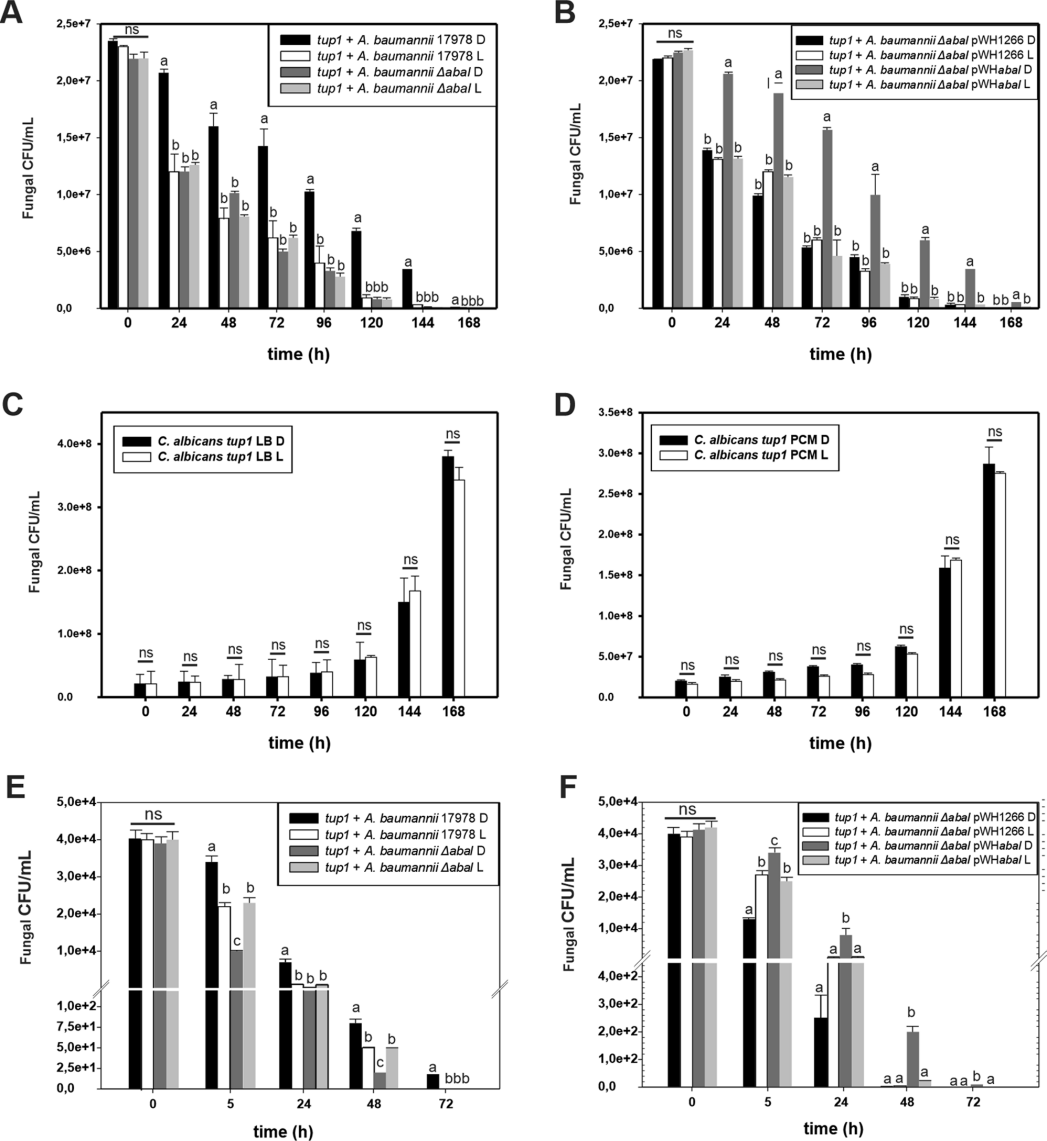

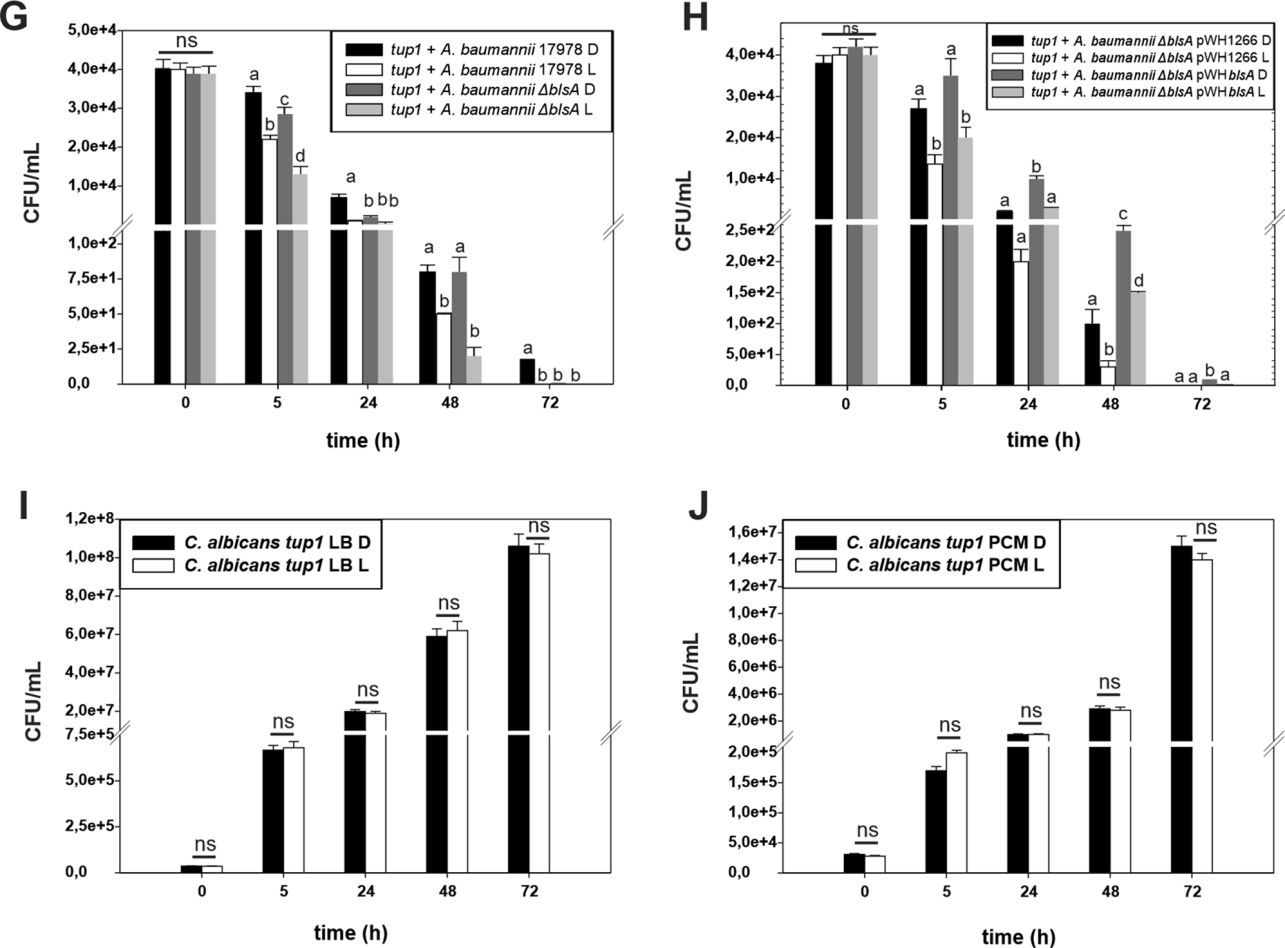
Killing of *tup1* mutant *C. albicans* by *A. baumannii* cells in response to light. (**A–D**) Killing of *tup1* mutant *C. albicans* by *A. baumannii* cells in response to light at environmental temperatures. (**A**) Fungal filaments were co-incubated with bacterial cells from the ATCC 17978 parental strain or the isogenic *∆abaI* mutants in presence of the corresponding filtered sterilized preconditioned medium under blue light (L) or in darkness (D) at 23 °C. (**B**) Similar to experiment shown in panel A but using the mutant harboring the empty pWH1266 plasmid or the complementing plasmid pWHAbaI. Aliquots were taken at different times and plated on selective media to counter select for *A. baumannii* and follow *C. albicans* survival (see “Methods” for details). Shown are the means and standard deviations from three replicates. Different letters indicate significant differences as determined by ANOVA followed by Tukey’s multiple comparison test (*p* < 0.05). Representative results from three independent experiments are shown in each case. (**C**,**D**) Controls performed by co-incubation of *C. albicans* alone in the presence of LB or PCM, respectively. (**E–J**) Killing of *tup1* mutant *C. albicans* by *A. baumannii* cells in response to light at 37 °C. Fungal filaments were co-incubated with bacterial cells from the ATCC 17978 parental strain and the *∆abaI* mutant (**E**) or the *∆blsA* mutant (**G**) in presence of the corresponding filtered sterilized preconditioned medium under blue light (L) or in darkness (D) at 37 °C. (**F**,**H**) Similar to experiment shown in panel (**E**,**G**) but using the mutants harboring the empty pWH1266 plasmid or the complementing plasmids pWHAbaI or pWHBlsA, respectively. Aliquots were taken at different times and plated on selective media to counterselect for *A. baumannii* and follow *C. albicans* survival (see “Methods” for details). Shown are the means and standard deviations from three replicates. Different letters indicate significant differences as determined by ANOVA followed by Tukey’s multiple comparison test (*p* < 0.05). Representative results from three independent experiments are shown in each case. (**I**,**J**) Controls performed by co-incubation of *C. albicans* alone in the presence of LB or PCM, respectively.

*C. albicans tup1* filaments recovery was significantly lower when co-incubated with *A. baumannii* ATCC 17978 under blue light than in the dark, showing that *A. baumannii* is more virulent against *C. albicans tup1* under illumination conditions (Fig. 4A), which is consistent with previous results^14^. Conversely, the number of *tup1* mutant filaments recovered when co-incubated with the *ΔabaI* mutant was similar between light and darkness, and also similar to that recovered when co-incubated with the wild type strain under blue light (Fig. 4A). Thus, the *ΔabaI* mutant lost the ability to photoregulate killing of *tup1* mutant filaments. The *ΔabaI* mutant harboring the empty pWH1266 plasmid behaved as the *ΔabaI* mutant (Fig. 4B). In contrast, the *ΔabaI* mutant harboring plasmid pWHAbaI restored the wild type phenotype showing increased virulence under blue light (Fig. 4B). As controls, no differences between light and darkness were detected in the number of *tup1*filaments recovered when incubated without bacteria in LB or in preconditioned media (PCM) (Fig. 4C,D, respectively), indicating that growth is not affected by blue light.

The overall results show that AbaI is involved in modulation by light of *A. baumannii*'s virulence when tested using the killing of *C. albicans tup1* mutant filaments model. AbaI is necessary for the lower virulence exhibited by *A. baumannii* in the dark (Fig. 4A,B), while the presence of BlsA, in turn, is involved in stimulation of virulence in the presence of light^14^.

### BlsA interacts with the regulator AbaR in the dark at moderate temperatures in *A. baumannii*

**Figure 5:**
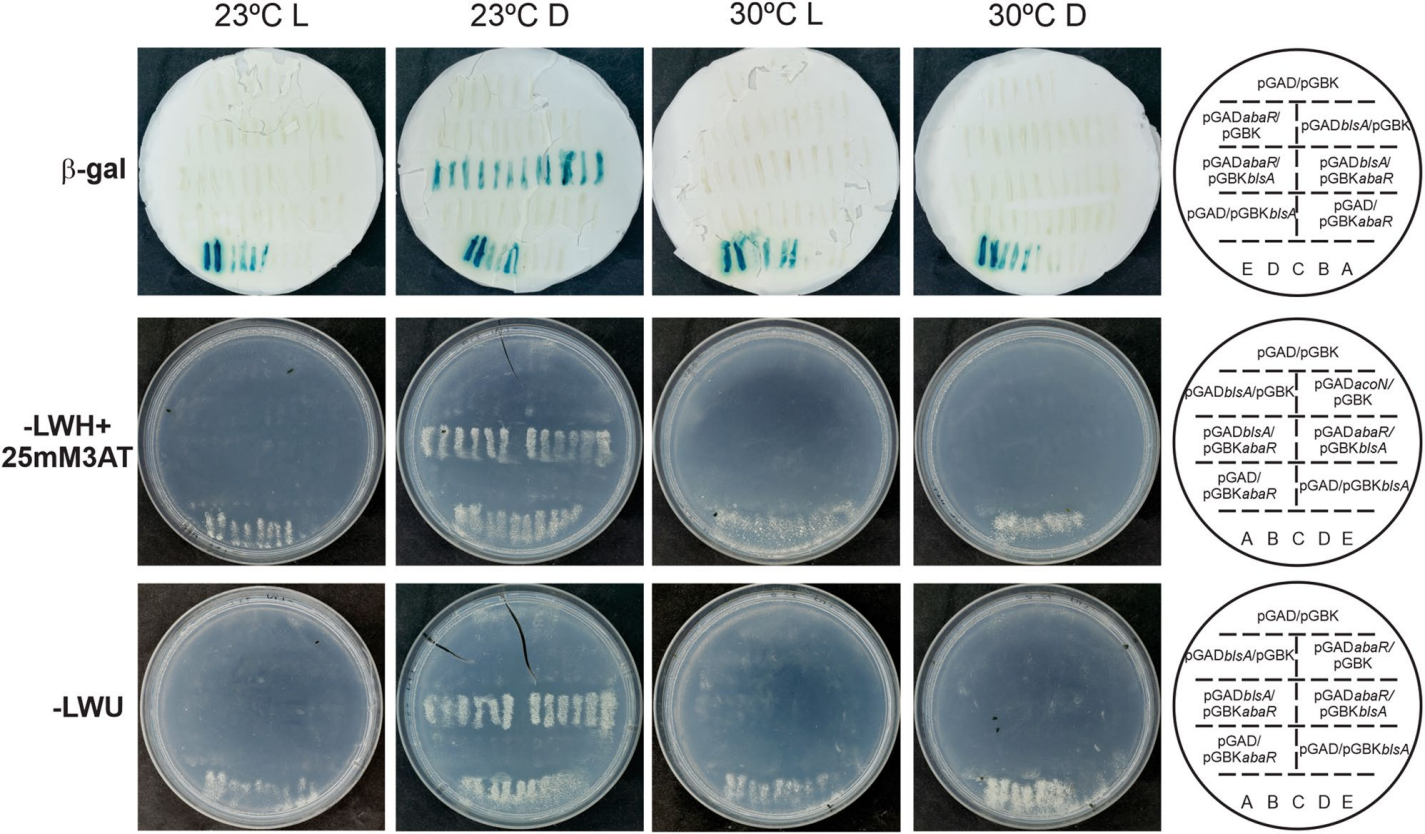
BlsA–AbaR interaction studied by Y2H assays. Six clones of MaV203/pGAD-*blsA* or MaV203/pGAD-*abaR* transformed with plasmids pGBK-*abaR* or pGBK-*blsA*, respectively, as well as plasmid pGBK-T7 or pGAD-T7 as negative control, were patched in each plate. Also included are reciprocal combinations, and self-activation as well as different strength interaction controls (strains A–E). The description and order of yeast streaks on each plate are indicated in the scheme on the right. Results for the *lacZ* reporter, the histidine auxotrophic marker and the uracil reporter are indicated in the top, middle and bottom panels. Experiments were performed in triplicate and representative results are shown.

To study whether BlsA interacts with AbaR, we performed yeast two-hybrid experiments using an adapted version of ProQuestTM Two-Hybrid System^20,21^. Strain Mav 203, which is included as host yeast in the system, harbors three reporter genes with different promoters: *lacZ* and HIS3 and URA3. His and URA3 solve the histidine and uracile MAV203 auxotrophies upon expression. Then, if BlsA and AbaR do interact, it would be expected the appearance of blue color as well as growth in the absence of histidine or uracil. pGAD-T7Gw and pGBK-T7Gw plasmids have been adapted to express *blsA* and *abaR*, as fusions to GAL4 DNA binding domain (DB) or activation domain (AD). Self-activation controls (pGAD-T7Gw and pGBK-T7Gw empty vectors) as well as different strength interaction controls (A–E) were also included. BlsA was previously shown to interact with different partners in an illumination and temperature-dependent manner^20,21^. Figure 5 shows results of Y2H assay experiments performed under different illumination and temperature conditions. BlsA-AbaR interaction was observed as the appearance of blue color and growth in SC defined media without the addition of histidine or uracil only at 23 °C in the dark (Fig. 5). Both pGAD*blsA*/pGBK*abaR* and pGAD*abaR*/pGBK*blsA* combinations generated positive signals, indicating that the interactions occurred independently of the host vector (Fig. 5). Also, absence of self-activation of each protein fused to DB or AD: (pGAD-T7/pGBK*blsA* or pGBK*abaR*) or (pGBK-T7/pGAD*blsA* or pGAD*abaR*) was verified in the corresponding controls (Fig. 5). On the contrary, no interactions were observed for AbaR and BlsA for any of the reporters tested at 23 °C in the presence of blue light, despite interaction controls behaved as expected (Fig. 5). Altogether, the data indicate that BlsA interacts with AbaR only in the dark at 23 °C, in a light-dependent manner.

At higher temperatures such as 30 °C, null or negligible BlsA–AbaR interactions were observed (Fig. 5), either in the dark nor under blue light.

### Light modulates the production of AHLs in *A. baumannii* at 37 °C

**Figure 6:**
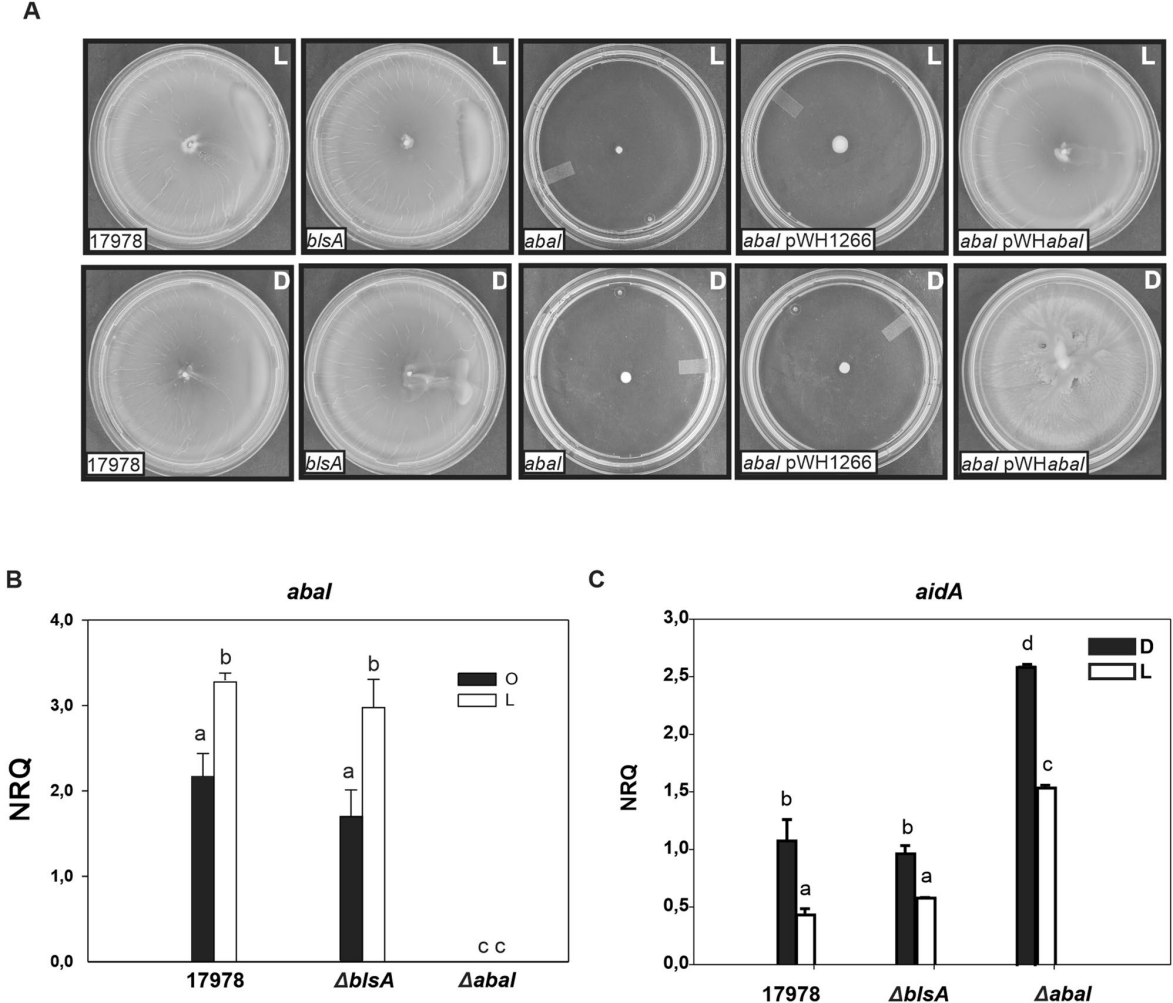
Contribution of AbaI to modulation by light of motility in *A. baumannii* at 37C. (**A**) Cells of the parental strain ATCC 17978, the isogenic *ΔblsA* and *ΔabaI* mutants, as well as this mutant harboring the empty pWH1266 plasmid or the *abaI*-complementing plasmid pWHAbaI were inoculated on the surface of motility plates. Plates were inspected and photographed after incubation in darkness (D) or in the presence of blue light (L) at 37 °C. Representative results of three independent experiments are shown. (**B**,**C**) *abaI* and *aidA* expression is modulated by light in *A. baumannii* at 37 °C. Estimation by qRT-PCR of the expression levels of the gene coding for an acyl homoserine lactone synthase, *abaI* (**B**), and the gene encoding a lactonase, *aidA* (**C**), in cells recovered from motility plates inoculated with ATCC 17978 wild-type, *∆blsA* and *∆abaI* incubated at 37 °C under blue light (L) or in the dark (D). The data shown are the mean and standard deviation of normalized relative quantities (NRQ). Different letters indicate significant differences as determined by ANOVA followed by Tukey’s multiple comparison test (*p* < 0.05). Representative results of three independent experiments are shown.

As has been previously reported, motility is not photoregulated in *A. baumannii* ATCC 17978 at 37 °C^14^ (Table 1; Fig. 6A), and neither is biofilm formation in glass (not shown). Motility is completely dependent on AbaI, both under blue light and in the dark (Table 1; Fig. 6A). β-Galactosidase activity was found to be enhanced when *A. tumefaciens* NT1 (pZLR4) was supplemented with supernatants recovered from motility plates incubated under blue light with respect to dark conditions at 37 °C, thus indicating the presence of higher amounts of AHLs under blue light (Table 1; Supplementary Figure S4A). Supernatants recovered from the *ΔblsA* mutant behaved as the wild type, i.e., generated enhanced β-galactosidase activity under blue light compared to dark conditions (Table 1; Supplementary Figure S4A). The *abaI* mutant as well as the *abaI* mutant harboring pWH1266, did not induce β-galactosidase activity, neither under blue light nor in the dark (Table 1; Supplementary Figure S4A). This is consistent with the absence of AHLs expected in the AHL synthase mutant. On the contrary, expression of *abaI* from pWH*abaI* in the *abaI* mutant background restored β-galactosidase activity, which was higher under blue light with respect to dark conditions. These results show that the production of AHLs is regulated by light in *A. baumannii* at 37 °C, in a BlsA-independent but AbaI-dependent manner.

Similar experiments with the biosensors *C. subtsugae* CV026 and *C. violaceum* VIR07 for short and long-chain AHLs, respectively, produced similar pattern as that observed for *A. tumefaciens* NT1 (pZLR4) (Table 1; Supplementary Figures S4B, S5A, respectively), confirming that the production of short as well as long-chain AHLs is regulated by light in motility in *A. baumannii* at 37 °C*,* in a BlsA-independent AbaI-dependent manner.

Finally, filtered-sterilized supernatants recovered from biofilm tubes incubated under blue light or in the dark at 37 °C were also used to supplement the *C. subtsugae* CV026 and *C. violaceum* VIR07 biosensors (Table 1; Supplementary Figure S6). Again, violacein production was found to be higher under blue light than in the dark in the ATCC 17978 wild type strain. Also, the derivative strains showed a similar pattern as that observed for supernatants recovered from motility plates (Table 1; Supplementary Figures S4, S5A). Overall, Table 1 and Supplementary Figure S6 indicate that the production of both short as well as long-chain AHLs is regulated by light in biofilms in *A. baumannii* at 37 °C*,* with increased AHL production under blue light compared to dark conditions, in a BlsA-independent AbaI-dependent manner.

It should be noted that at 37 °C, the production of AHLs is higher under blue light than in the dark both for motility as well as biofilm formation, while the opposite occurs at 23 °C, i.e., AHLs production is higher in the dark than under blue light. In addition, the magnitude of the light–dark difference is much higher at 23 °C than at 37 °C.

### Quorum quenching activity is stimulated in the dark at 37 °C in *A. baumannii*

We evaluated here whether the supernatants of sonicated cells recovered from motility plates incubated under blue light or in the dark at 37 °C presented quorum quenching activity. For this purpose, these supernatants were incubated in the presence of 2 µg of standard for 6 h, and then the mixture was loaded on a central well generated in a biosensor*-*inoculated LB plate. The extent of inhibition of violacein production compared to the control without supernatant provides an indication of quorum quenching activity.

When the *C. subtsugae* CV026 biosensor and the C8-AHL standard were used, quorum quenching activity was observed for supernatants incubated both under blue light and in the dark for all the strains studied, i.e. the wild type and the ∆*blsA* and ∆*abaI* mutants. Quorum quenching activity was only slightly higher in the dark than under blue light for all the strains included (Table 1; Supplementary Figure S5B). On the contrary, when the *C. violaceum* VIR07 biosensor and the C10-AHL standard were used, quorum quenching activity was much pronounced in the dark compared to illumination conditions for all the strains studied (Table 1; Supplementary Figure S5C). These results show the presence of quorum quenching activity modulated by light in *A. baumannii* at 37 °C, in a BlsA and AbaI-independent manner.

### *abaI* and *aidA* expression are modulated by light at 37 °C

We studied next the expression of *abaI* in cells recovered from motility plates incubated under blue light or in the dark at 37 °C. Figure 6B shows that expression of *abaI* is approximately 1.5 folds higher under blue light than in the dark, indicating that it is modulated by light in ATCC 17978 at 37 °C. The *Δbls*A mutant behaved as the wild type strain, showing that modulation by light of *abaI* expression is independent of BlsA (Fig. 6B). As expected, expression of *abaI* in the *ΔabaI* mutant was null or negligible (Fig. 6B). The overall results show that the expression of *abaI* is modulated by light at 37 °C, being induced under blue light in a BlsA-independent manner. This result is consistent with the higher production of AHLs under blue light in ATCC 17978 at 37 °C. It is also worth mentioning the photorregulation of *abaI* expression and AHL production at 37 °C is opposite with respect to that observed 23 °C, as they are induced in the dark at 23 °C. In addition, the magnitude of the differences in *abaI* expression and AHL production between light and darkness were much higher at 23 than at 37 °C.

Interestingly, *aidA* expression pattern was inverse to that of *abaI*. In particular, *aidA* expression was induced approximately 2 folds in the dark compared to illumination conditions at 37 °C (Fig. 6C). The *blsA* mutant behaved as the wild type, while the *abaI* mutant presented increased levels of expression both under blue light and in the dark than the wild type, while maintaining a difference of 1.5 folds between light and darkness (Fig. 6C). This result is consistent with the higher quorum quenching activity detected in the dark in ATCC 17978 at 37 °C, as well as with the lower amounts of AHL observed in this condition.

### Detection of Acyl-HSLs by TLC and GC-mass spectrometry at 37 °C

When AHL extracts obtained from biofilms supernatants of the wild type *A. baumannii* ATCC 17978 strain, and its isogenic mutant *abaI* harboring the pWHAbaI plasmid grown under blue light and in the dark at 37 °C were separated and analyzed by TLC developed with *C. subtsugae* CV026, the presence of higher amounts of AHLs under blue light were detected respect to darkness (Fig. 3B). The spots observed are compatible with C8-AHL standard. When equivalent TLCs were visualized with the *C. violaceum* VIR07 reporter for long-chain AHLs (Fig. 3B), two main spots compatible with C8-AHL, C10-AHL present in higher amounts under blue light respect to dark conditions were observed. Again, TLCs results were consistent with biosensor assays.

Figure 3D shows the amounts of each of the different AHLs identified and normalized to the quantity measured at 37 °C blue light (L) condition, determined by GC–MS. As is observed in Fig. 3D, C8–C10 and C12-AHLs, as well as 3OHC12-AHL were detected at 37 °C. Interestingly, and consistently with the biosensor assays as well as with TLCs, the presence of C8, C10, C12 as well as 3OHC12-AHLs was detected in higher amounts under blue light respect to dark conditions both for the wild type as well as for the *abaI* mutant harboring the pWHAbaI plasmid (Fig. 3D).

### Light modulates *A. baumannii*'s virulence against *C. albicans* at 37 °C through AbaI

*C. albicans tup1* filaments recovery was lower when co-incubated with *A. baumannii* ATCC 17978 under blue light than in the dark at 37 °C, showing that *A. baumannii* is more virulent against *C. albicans tup1* under blue light (Fig. 4E), as occurs at environmental temperatures (Fig. 4A). Conversely, the *ΔabaI* mutant showed an opposite pattern of photoregulation, with less *C. albicans tup1* filaments recovery in the dark than under blue light (Fig. 4E), indicating that the ability to kill *C. albicans tup1* is enhanced in the dark in this mutant. The *ΔabaI* mutant harboring the empty pWH1266 plasmid behaved as the *ΔabaI* mutant (Fig. 4F). In contrast, the *ΔabaI* mutant harboring plasmid pWHAbaI, which expresses a wild type copy of *abaI* directed by its own promoter, restored the wild type phenotype showing increased virulence under blue light (Fig. 4F). *ΔblsA* behaved similarly as the wild type, suggesting that it is not involved in this response to light at 37 °C (Fig. 4G,H). As controls, *C. albicans tup1* filaments recovery was similar between blue light and in the dark both in LB and in (PCM) (Fig. 4I,J, respectively), indicating that growth is not affected by blue light. From the above results, it follows that in the absence of AbaI the bacteria display a virulence mechanism modulated by light at 37 °C, in which virulence is enhanced in the dark instead than under blue light, as occurs in a wild type background. Overall, AbaI is involved in modulation by light of *A. baumannii*'s virulence against *C. albicans tup1*at temperatures found in warm-blooded hosts.

## Discussion

In this work, we provide strong evidence indicating that light directly modulates the quorum network in *A. baumannii*. We focused on motility and biofilms formation, which are different bacterial processes shown to depend on quorum sensing^4–7^, and are modulated by light^14^. As has been extensively described, our results also show that AbaI is involved in these responses. Interestingly, complementation of the *∆abaI* mutant with *abaI* expressed from a plasmid restored photoregulation of motility and biofilm formation at moderate temperatures suggesting that AbaI is a direct or indirect component of the light signaling cascade. The production of AHL was found to be modulated by light on cells recovered both from motility and biofilm assays at moderate temperatures, as determined by the use of the *A. tumefaciens* NT1 (pZLR4), *C. subtsugae* CV026 and *C. violaceum* VIR07 biosensors, in a BlsA and AbaI-dependent manner. In fact, AHL production followed motility and biofilm formation patterns in the different strains. In agreement, *abaI* expression was found to be stimulated in the dark, which followed AHL production. Surprisingly, despite significant and similar levels of AHLs were produced in the *blsA* mutant under blue light and in the dark, expression of *abaI* was low and similar to the wild type under blue light in this mutant, showing no correlation between *abaI* expression, motility and production of AHLs at environmental temperatures. A possible explanation for these results could be that BlsA modulates the “timing” of *abaI* expression. Then, *abaI* expression could have been significant in the *blsA* mutant both under blue light and in the dark in a time lapse previous to sample recovery, allowing thus AHLs production and motility, and could have decreased reaching minimal expression latter, which is typical of the expression of AHL synthase genes. Quorum quenching activity was found to be higher under blue light than in the dark at environmental temperatures, in a BlsA dependent-manner. Consistently, expression patterns of *aidA,* reported to function as a lactonase^30^, are also in agreement with the AHL pattern under blue light and darkness. In particular, *aidA* expression was found to be induced under blue light at environmental temperatures, which is consistent with the null or reduced presence of AHLs and increased quorum quenching activity detected in this condition. TLCs and GC–MS confirmed the presence of the short as well as long chain AHLs C8-, C10 and C12-AHL in higher levels in the dark respect to blue light in biofilms at 23 °C. No hydroxylated forms were detected, despite 3OHC12-AHLs has been reported to be the main AHLs in *A. baumannii* at 37 °C^10,32,33^. In fact, different conditions such as temperature and culture medium have been shown to modulate AHL production^33–35^. At temperatures compatible with warm-blooded hosts such as 37 °C, modulation of the quorum network by light was also observed. However, the pattern was opposite to that observed at environmental temperatures. In fact, AHL production was higher under blue light than in the dark in a BlsA-independent AbaI-dependent manner. Despite the presence of 3OHC12-AHL was detected, this was not the predominant AHL in the biofilm culture conditions tested (not shown). In addition, *aidA* expression was found to be induced in the dark, which is consistent with less AHL production and higher quorum quenching activity at this condition. It should be noted that the net amount of AHLs at each condition is the result of the contribution of the activities of AbaI, AidA as well as other putative lactonases or quorum quenching molecules not yet described or studied in the present work. Overall, we show that there is a fine tuning of quorum sensing vs. quenching activities dictated by the influence of light on expression of AHLs synthases and lactonases and integrating also a temperature signal, which results in differential production of AHLs in response to illumination and temperature.

**Figure 7:**
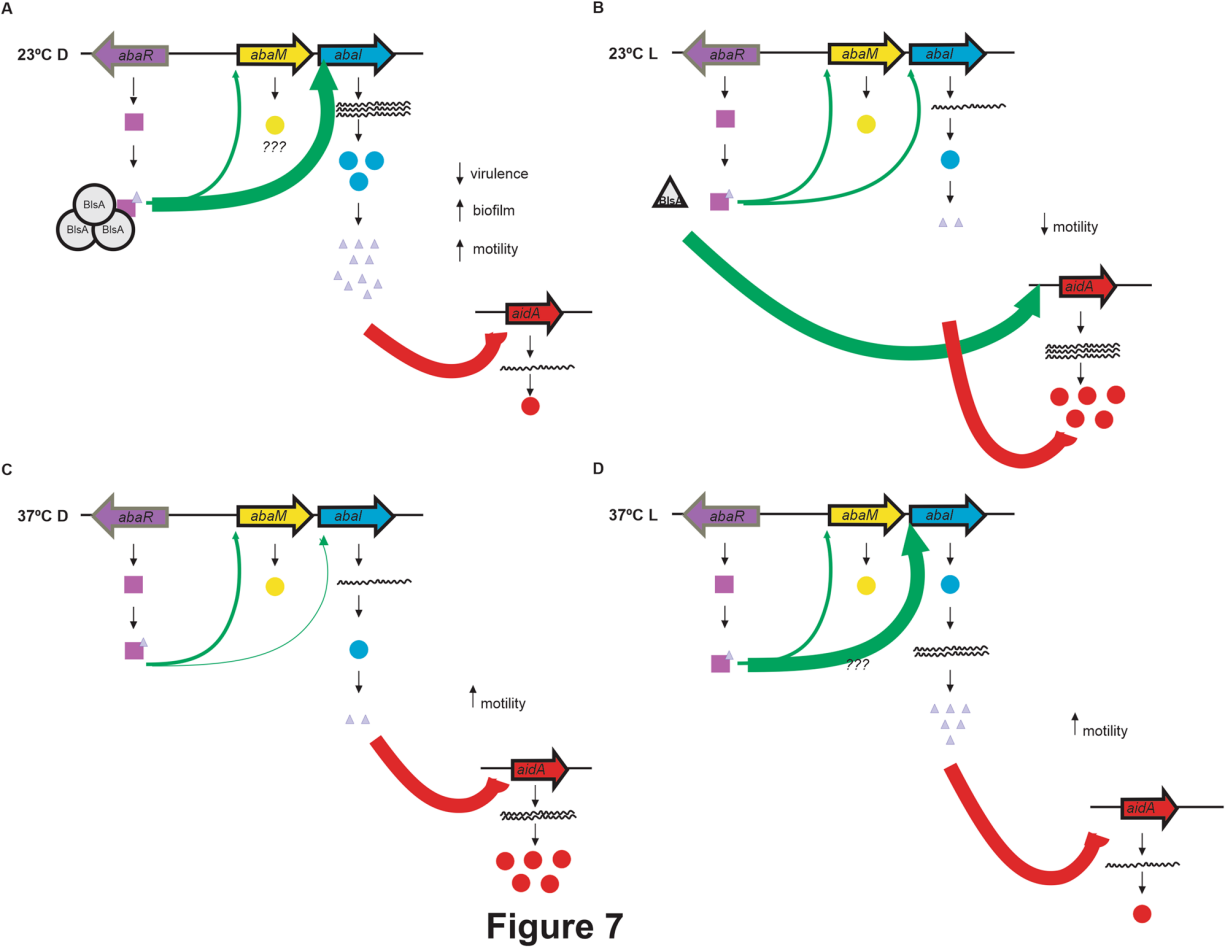
Working model depicting current knowledge regarding modulation by light of quorum sensing in *A. baumannii*. At environmental temperatures such as 23 °C, the photoreceptor BlsA interacts with AbaR only in the dark most probably with the bound AHL, inducing expression of the *abaI* gene and the production of AHL at this condition. At environmental temperatures such as 23 °C but in the presence of light, BlsA does not interact with AbaR, most likely because it is in a non-permissive conformation. Interestingly, BlsA induces of expression of AidA, reported as a lactonase, in the in the presence of light but not in the dark. This is consistent with the increased quorum quenching activity observed in the presence of light, which is even more deepened in the *ΔabaI* mutant. The overall result is the presence of higher levels of AHL in the dark than under blue light (**A**,**B**). At 30 °C, and therefore we infer that neither does it at 37 °C, BlsA does not interact with AbaR, neither in the dark, nor under blue light (**C**,**D**), indicating that this photorreceptor is not involved in the quorum response at this temperature. *abaI* expression levels, as well as AHLs are higher in the presence of light than in the dark. Interestingly, *aidA* expression levels were induced in the dark and significantly increased in the *abaI* mutant both in the dark and under blue light indicating that AbaI or its products inhibit expression of the *aidA* lactonase. However, this was not reflected in a higher quorum quenching activity in the *abaI* mutant.

Figure 7 summarizes the working model depicting light modulation of the *A. baumannii* QN based on results obtained in this work^10^. At environmental temperatures such as 23 °C, the photoreceptor BlsA interacts with AbaR only in the dark most probably with the bound AHL, inducing expression of the *abaI* gene and the production of AHL at this condition (Fig. 7A). At environmental temperatures such as 23 °C but in the presence of light, BlsA does not interact with AbaR, most likely because it is in a non-permissive conformation (Fig. 7B). Interestingly, BlsA induces of expression of the AidA, reported as a lactonase, in the in the presence of light but not in the dark. This is consistent with the increased quorum quenching activity observed in the presence of light, which is even more deepened in the *ΔabaI* mutant. The overall result is the presence of higher levels of AHL in the dark than under blue light, which correlates with higher motility (Fig. 7A,B).

At 37 °C, BlsA does not interact with AbaR, neither in the dark, nor under blue light, indicating that this photorreceptor is not involved in the quorum response at this temperature. *abaI* expression levels, as well as AHLs are higher in the presence of light than in the dark. Interestingly, *aidA* expression levels were induced in the dark and significantly increased in the *abaI* mutant both in the dark and under blue light, indicating that AbaI or its products inhibit expression of the *aidA* lactonase.

Moreover, we show that AbaI contributes to modulation of *A. baumannii*'s virulence by light, reducing *C. albicans* killing in the dark respect to light conditions both at environmental temperatures as well as temperatures found in warm-blooded hosts. These results are in agreement with others' indicating that the mutation of the AbaI synthase significantly reduces virulence in in vivo models^36,37^.

Integration of quorum and light signals to modulate collective behaviors in *P. aeruginosa* have been recently reported^38^. In particular, the response regulator AlgB has been shown to be the node that integrates three inputs: quorum sensing through the activating action of the quorum sensing receptor RhlR, light through the BphP photorecepor, and an unknown signal via its partner KinB, to modulate biofilm formation and virulence^31^. In this work, we show that light directly modulates the quorum network. Evidence of this includes the interaction of the photoreceptor BlsA with AbaR in the dark but not in the presence of blue light at environmental temperatures. Also, expression of *abaI* and *aidA* are modulated by light, and finally the presence of AHL is differential depending on the illumination conditions. Thus, a new concept is introduced in this work with advances on mechanistical insights. However, further work is still required to completely elucidate the mechanism of light regulation of quorum sensing in *A. baumannii,* which surely involves many other effectors. For example, the role of AbaM in the model has yet to be established, as it may also participate directly in modulation of the response to light and likely interact with BlsA.

## Methods

### Bacterial strains, plasmids, and media

Bacterial strains and plasmids used in this work are listed in Table 2. Luria–Bertani (LB) broth (Difco) and agar (Difco) were used to grow and maintain bacterial strains. Broth cultures were incubated at the indicated temperatures either statically or with shaking at 200 rpm.

**Table 2:** Bacterial, yeast strains and plasmids used in this study.

Strain/plasmid	Relevant characteristic	Source or reference
***A. baumannii***		
ATCC 17978	Clinical isolate	ATCC
ATCC 17978 *ΔabaI*	Generated by mutagenesis using plasmid pMO130-telR	^43^
ATCC 17978 *ΔblsA*	*blsA*::aph derivative of 17978; Km^r^	^14^
ATCC 17978 *ΔblsA* pWHBlsA	17978 *ΔblsA* harboring plasmid pWH*blsA*; Km^R^ Amp^R^	^14^
ATCC 17978 *ΔblsA* pWH1266	17978 *ΔblsA* harboring pWH1266; Km^R^ Tet^R^ Amp^R^	^14^
ATCC 17978 *ΔabaI* pWHAbaI	17978 *ΔabaI* harboring plasmid pWH*abaI*; Km^R^ Amp^R^	This study
ATCC 17978 *ΔabaI* pWH1266	17,979 *ΔabaI* harboring plasmid pWH1266; Km^R^ Tet^R^ Amp^R^	This study
***Chromobacterium subtsugae***		
CV026	AHLs short chain biosensor, *cvi*I::mini-Tn5 KnR	^28^ ^42^
***Chromobacterium violaceum***		
VIR07	AHLs long chain biosensor *cvi*I mutant	^29^
***E. coli***		
DH5α	Used for DNA recombinant methods	Gibco-BRL
***Saccharomyces cerevisiae***		
Mav 203 strain	MATa, *leu*2-3,112, *trp*1-901, *his*3-D200, *ade*2-101, *gal*4D, *gal*80D, SPAL10::URA3, GAL1::*lac*Z, HIS3UAS GAL1::HIS3, YS2, *can*1R and *cyh*2R	Thermofisher
**Plasmids**		
pBluescript	PCR cloning vector; Ampr	Promega
pWH1266	*E. coli-A. baumannii* shuttle vector; Amp^R^ Tet^R^	^44^
pWHBlsA	pWH1266 harboring wildtype copy of *blsA* from ATCC 17978 expressed under its own promoter; Amp^R^	^14^
pWHAbaI	pWH1266 harboring wild-type copy of *abaI* from ATCC 17978 expressed under its own promoter; Amp^R^	This study
pENTR3C	Gateway system entry-vector	Invitrogen- Thermofisher
PGAD-T7-GW	Y2H AD-fusion vector, adapted to Gateway System	Clontech, ^39^
PGBK-T7-GW	Y2H DB-fusion vector, adapted to Gateway System	Clontech, ^39^

### Plasmid construction

#### Y2H

PCR amplifications of *blsA* and *acoR* coding sequences were performed from *A. baumannii* ATCC 17978 genomic DNA using primers *blsAdh*^21^ and *abaRdh* (Table 3). The amplification products were subsequently cloned into the BamHI and XhoI sites of Gateway entry vector pENTR3C (Invitrogen) (Table 1). The cloned fragments were then transferred to pGBKT7-Gw and pGADT7-Gw Y2H vectors (Clontech) by using LR Clonase^20,21,39^. In the yeast host, these plasmids express the cloned coding sequences as fusion proteins to the GAL4 DNA-binding domain (DB) or activation domain (AD), respectively, under the control of the constitutive ADH1 promoter. Automated DNA sequencing confirmed correct construction of each plasmid.

**Table 3:** Primers used in this study.

Name	SEQUENCE (5′–3′)	References
*abaRdhF*	GGATCCATGGAAAGTTGGCAAGAAGATTT	This study
*abaRdhR*	CTCGAGACCTACAAAAGCCCTAGCATTACAG	This study
*blsAdhF*	GGATCCATGAACGTTCGCCTGTGT	^20^
*blsAdhR*	CTCGAGTGCTAGAACGGGTTTACTC	^20^
*pabaIF*	GGATCCTACAAGTGCTTCCACTTATTTTTCA	This study
*pabaIR*	GGATCCTTTCTTATATAGGACTCATGCCT	This study
*aidAF*	GGGAACTTCTTTCGGTGGAG	This study
*aidAR*	AACAGCAGCAAGTCGATTATCA	This study
*abaIF*	CCGCTACAGGGTATTTGTTGAAT	This study
*abaIR*	GCAGGGAATAGGCATTCCATTG	This study
*rpoBF*	CAGAAGTCACGCGAAGTTGAAGGT	^17^
*rpoBR*	AACAGCACGCTCAACACGAACT	^17^
*recAF*	TACAGAAAGCTGGTGCATGG	^14^
*recAR*	TGCACCATTTGTGCCTGTAG	^14^

**Table 1:** Summary of AHLs detection using the different biosensors.

	17978 L	17978 D	*∆blsA* L	*∆blsA* D	*ΔblsA* pWH1266 L	*ΔblsA* pWH1266 D	*ΔblsA* pWH*blsA* L	*ΔblsA* pWH*blsA* D	*ΔabaI* L	*ΔabaI* D	*ΔabaI* pWH1266 L	*ΔabaI* pWH1266 D	*ΔabaI* pWH*abaI* L	*ΔabaI* pWH*abaI* D
Motility CV026 23 °C	−	++	++	++	++	++	−	+++	−	−	−	−	−	+++
Motility VIR07 23 °C	−	+	+	+	+	+	−	++	−	−	−	−	−	++
Motility NT1 (pZLR4) 23 °C	−	++	++	++	++	++	−	++	−	−	−	−	−	++
Biofilms CV026 23 °C	−	++	++	++	++	++	−	+++	−	−	−	−	−	+++
Biofilms VIR07 23 °C	−	+	+	+	+	+	−	++	−	−	−	−	−	++
Motility QQ CV026 23 °C	+	++	++	++	ND	ND	ND	ND	−	++	ND	ND	ND	ND
Motility QQ VIR07 23 °C	+/−	+	+	+	ND	ND	ND	ND	−	+	ND	ND	ND	ND
Motility CV026 37 °C	+++	++	+++	++	ND	ND	ND	ND	−	−	−	−	+++	++
Motility VIR07 37 °C	++	+	++	+	ND	ND	ND	ND	−	−	−	−	++	+
Motility NT1 (pZLR4) 37 °C	++	+	++	+	ND	ND	ND	ND	−	−	−	−	+++	+
Biofilms CV026 37 °C	+++	+	+++	+	ND	ND	ND	ND	−	−	−	−	+++	+
Biofilms VIR07 37 °C	++	+	++	+	ND	ND	ND	ND	−	−	−	−	++	+
Motility QQ CV026 37 °C	++	++/−	++	++/−	ND	ND	ND	ND	++	++/−	ND	ND	ND	ND
Motility QQ VIR07 37 °C	++/−	+/−	++/−	+/−	ND	ND	ND	ND	++/−	+/−	ND	ND	ND	ND

*ND* non-determined. (−) no pigment production. The amount of + correlates with the level of pigment production.

#### pWHAbaI

*abaI* coding sequence and its promoter region were amplified by PCR using *A. baumannii* ATCC 17978 genomic DNA as template and primers p*abaI*F and p*abaI*R (Table 3), which contained BamHI restriction site tails. The amplification product was cloned into pWH1266 through the BamHI site, following protocols described in Mussi et al*.*^14^. Automated DNA sequencing confirmed the proper construction of pWHAbaI plasmid.

### Blue light treatments

Blue light treatments were conducted as reported before^14,15,17,20–22,40^. Briefly, cells were grown in the dark or under blue light emitted by an array composed of 3 × 3-LED module strips emitting an intensity of 6–10 mol photons/m^2^/s, with emission peaks centered at 462 nm^14^.

### Yeast two-hybrid (Y2H) assays

Yeast two-hybrid experiments were conducted following procedures described before^20,21,39^. *Saccharomyces cerevisiae* Mav 203 strain (MATα, *leu*2-3,112, *trp*1-901, *his*3-Δ200, *ade*2-101, *gal*4Δ, *gal*80Δ, SPAL10::URA3, GAL1::*lac*Z, HIS3UAS GAL1::HIS3, LYS2, *can*1R, and *cyh*2R) was transformed with the different expression vectors. First, BlsA and AbaR were analyzed for self-activation. For this purpose, MaV203 yeast strain containing the pGAD-T7 empty vector was transformed with the DNA DB-fusion protein expressing vectors (pGBK-X) (X = BlsA or AbaR). Conversely, MaV203 yeast strain containing the pGBK-T7 empty vector was then transformed with the AD-fusion protein expressing vectors (pGAD-Y) (Y = BlsA or AbaR). In addition, these strains were used for determination of the optimal 3-amino-1,2,4-triazole (3AT) concentration required to titrate basal HIS3 expression. MaV203/pGBK-X strains were afterward transformed with each pGAD-Y plasmids. Transformations using one or both Y2H plasmids were performed by the lithium acetate/single-stranded carrier DNA/polyethylene glycol method described in Gietz and Woods^45^, and plated in convenient minimal selective medium [synthetic complete (SC) medium without leucine (-leu) for pGAD-Y transformants, SC without tryptophan (-trp) for pGBK-X transformants, and SC-leu-trp transformants carrying both plasmids]. The plates were then incubated at 30 °C for 72 h to allow growth of transformants. A “Master Plate” was then prepared using SC-leu-trp media, in which we patched: four to six clones of each pGBK-X/pGAD-Y containing yeasts, four to six self-activation control clones pGBK-X/pGAD and pGBK/pGAD-Y (Y DNA-binding negative control), and two isolated colonies of each of the five yeast control strains (A–E). The plates were incubated for 48–72 h at 23 °C in the dark. This Master Plate was then replica plated to SC–leu–trp–hisC 3AT and to SC–leu–trp–ura to test for growth in the absence of histidine (his) and uracil (ura), respectively (*his3* and *ura3* reporter activation), under the different conditions analyzed, i.e., dark/light; 23/30 °C, for at least 72 h. For development of blue color as a result of β -galactosidase (β-Gal) expression, transformed yeasts were replica plated on a nitrocellulose filter on top of a YPAD medium plate and grown at the different conditions (dark/light; 23/30 °C). Then, the cells on the nitrocellulose filter were permeabilized with liquid nitrogen and soaked in X-Gal solution (5-bromo- 4-chloro-3-indolyl-b-d-galactopyranoside in Z buffer (60 mM Na_2_HPO_4_, 40 mM NaH_2_PO_4_, 10 mM KCl, 1 mM MgSO_4_, pH 7.0), for 24 h at 37 °C following the manufacturer's recommendations (Invitrogen).

#### Analyses of gene expression by qRT-PCR

Retrotranscription and qRT-PCR analysis were done as described in Tuttobene et al.^21^, using primers listed in Table 3. Data are presented as NRQ (Normalized relative quantities) calculated by the qBASE method^41^, using *recA* and *rpoB* genes as normalizers.

#### Cell motility assay

Cell motility was tested on swimming agarose media: 1% tryptone, 0.5% NaCl and 0.3% agarose plates inoculated on the surface by depositing 3 µl of LB cultures grown to an optical density at 660 nm (OD_660_) of 0.3. The plates were incubated 24 or 48 h in the presence or absence of blue light at 37 °C or 23 °C, respectively. For AHL detection assays, the aqueous media containing the cells on the surface of motility plates was recovered, homogenized, and the optical density at 660 nm was determined. Cultures were then centrifuged, filtered-sterilized and amounts normalized to OD_660_ = 1.5 were loaded on biosensor plates, as is indicated in section AHLs detection using biosensors. Triplicate assays were done using fresh samples each time.

#### Biofilm formation assays

For biofilm assays, two milliliters of fresh swimming agarose broth medium contained into glass tubes were inoculated with 0.01 ml of an overnight shaking culture grown at 37 °C. The cultures were then incubated stagnantly at 23 °C or 37 °C either in darkness or under blue light for 48 or 24 h, respectively. Biofilms and pellicles that formed on the walls of the glass tubes were detected by visual inspection^14^. Replicate tubes were homogenized by vortexing and the cell density was determined by measurement of OD_660._ In general, no significant differences were registered between the different strains incubated under blue light vs. dark conditions, at each temperature. Nonetheless, whether any difference in optical density appeared, the amount of supernatants was normalized to OD_660_ = 0.5. Each culture was then centrifuged, and the supernatants were filter-sterilized and used for AHL detection, as indicated in the following item. Triplicate assays were done using fresh samples each time.

#### AHLs detection using biosensors

*Agrobacterium tumefaciens* NT1 (pZLR4), *C. subtsugae* (formerly *C. violaceum*) ATCC 31532 CV026^42^, and *C. violaceum* ATCC 12472 VIR07^29^, were used to detect the presence of AHLs in *A. baumannii* cultures recovered from motility plates or biofilm tubes. The *A. tumefaciens* NT1 (pZLR4) AHL biosensor, which contains a plasmid-localized traG-lacZ fusion (pZLR4)^25,26^, responds to AHLs of chain lengths ranging from C6 to C12^27^. *C. subtsugae cviI* mutant CV026^28^ does not produce AHLs, but induces the CviR upon exposure to exogenous short-chain AHLs, which results in rapid synthesis of a visually clear purple pigmentation, violacein. Particularly, violacein is inducible by compounds with N-acyl side chains from C_4_, to C_8_, in length, with varying degrees of sensitivity^28^. Violacein production in *C. violaceum* ATCC 12472 *cviI* mutant VIR07 is induced in response to the long-chain AHLs (C10–C16)^29^. Supernatants recovered from motility plates or biofilm tubes (approximately 500 µl), were loaded in a central well of LB plates previously inoculated with *C. subtsugae* CV026 or *C. violaceum* VIR07. *Chromobacterium* inoculation was performed by addition of 500 µL of overnight cultures grown to an OD_660_ = 2.5–5 ml of 0.7% agar media, which was then incorporated on top of the 1.5% agar LB plates. The plates were then incubated at 30 °C in the dark for 24 h.

#### AHL quorum quenching activity assay

Cells recovered from motility plates of the indicated strains incubated under blue light or in the dark at 23 °C or 37 °C for 48 or 24 h respectively, were normalized to OD_660_ = 1.5. The cells were recovered by centrifugation at 3000 g for 10 min and resuspended in 500 μl in swimming media (1% tryptone, 0.5% NaCl). The cells were sonicated and then centrifuged. Post-sonication supernatants were incubated with 2 µg of commercial standards, C8-AHL or C10-AHL, for 6 h in a shaker at 37 °C. Aliquots (500 μl) of the resulting supernatant were used to detect AHL degradation in a well diffusion assay in double agar plates, in which *C. subtsugae* CV026 or *C. violaceum* VIR07 were added to soft agar to detect inhibition of violacein^30^. Quantification of violacein production in the different strains was determined by measuring the area and integrated density of each complete plate and subtracting the corresponding values measured in the negative control, using ImageJ software (NIH). The values were normalized to the positive control, which received the arbitrary value of 100. The data shown are the means of three independent experiments, and error bars represent the standard deviation of the mean.

#### AHL extraction from culture supernatants

Extracts for analytical TLC were prepared from 10-ml cultures (2 tubes of 5 ml each) grown in swimming media pH 7 under blue light or in the dark at 23 °C for 48 h or 37 °C for 24 h. The tubes were then vortexed to disrupt biofilms and the OD_660_ was measured. No significant differences in OD_660_ were detected between the samples. Bacteria were then removed by centrifugation for 5 min at 5000 rpm and the pH was checked, and confirmed to be to be 7 ± 0.1 for all the samples analyzed. Then, the supernatants were filtered-sterilized and extracted twice with equal volumes of ethyl acetate acidified with 0.1% acetic acid. The organic phase was evaporated under N_2_ flow.

#### Analytical TLC

Procedures followed those described in Shaw et al.^27^. Extracted AHL samples were dissolved in 20 µl HPLC-grade acetonitrile and 2 µl were applied to C_18_ TLC plates (RP-18 F254 S, Merck) and developed with 60% methanol. After development, the solvent was dried, and plates were overlaid with a 0.75% soft agar layer seeded with an overnight culture of *C. subtsugae* CV026 or *C. violaceum* VIR07. After the agar solidified, the coated plates were incubated at 28 °C for 18 h.

#### GC-mass spectrometry

AHL extracts were dissolved in 100 µl HPLC-grade acetonitrile. For silylation, 100 μl of a mixture of 99% bis(trimethylsilyl)-trifluoro-acetamide (BSTFA) and 1% trimethylchlorosilane (TMCS) (Catalog Number 15238, Sigma-Aldrich, USA) was added. After purged with nitrogen, the mixture was allowed to react for 1 h at 80 °C. One μl of extracted sample was injected (Split 1:10) into the gas chromatograph-mass spectrometer Agilent 7890B gas chromatograph coupled to an Agilent 5977 A mass spectrometer (Agilent Technologies Inc., Palo Alto, CA, USA) and a 30 m HP-5 ms Ultra Inert with a 0.25 mm inner diameter and 0.25 μm film thicknesses (Agilent Technologies Inc., Palo Alto, CA, USA). The sample was injected at 250 °C with a gas flow rate of 1 ml/min. The temperature program was isothermal for 5 min at 70 °C, followed by a 5 °C/min ramp to 240 °C and a hold at 240 °C for 2 min. The total running time was 41 min. Ions were generated by a ionization voltage of 70 eV with a scan range of 50–600 Da.

Components of each of the GC peaks were identified by comparison of retention indexes and mass spectra data in the NIST 2011 Mass Spectral Library and also with an in-house database created with standards. Standard mixtures of C8, C10 and C12-AHL as well as 3OHC8, 3OHC10 and 3OHC12-AHL were also analyzed by GC–MS demonstrating that mass fragmentation patterns are unique to each AHL. Areas of ion chromatograms were obtained taking into account that the quantitation ion and the confirming ion are unique to that acyl homoserine lactones retention times.

#### Killing of *C. albicans* filaments

Assays were performed as described before^14,22^, with the modification of incubating 1-ml of the co-cultures without shaking at 37 °C from 24 to 72 h under dark or blue light conditions. Fungal CFU counts per ml were determined at each time point studied by plating convenient dilutions of the co-cultures on yeast extract-peptone-dextrose (YPD) agar containing 60 mg/ml tetracycline, 30 mg/ml chloramphenicol, and 30 mg/ml gentamicin, following incubation at 28 °C for 48 h.

### Statistical analysis

Experiments were performed in technical and biological triplicates and ANOVA followed by Tukey’s multiple-comparison test (P < 0.05) statistical analyses were performed using GraphPad Prism (GraphPad software, San Diego, CA, USA).

## Supplementary Information

Supplementary Information 1.

Supplementary Information 2.

Supplementary Information 3.

Supplementary Information 4.

Supplementary Information 5.

Supplementary Information 6.
